# Isolation, identification, and antibacterial evaluation of endophytic fungi from Gannan navel orange

**DOI:** 10.3389/fmicb.2023.1172629

**Published:** 2023-06-15

**Authors:** Huan Wang, Ziyue Liu, Fangfang Duan, Yan Chen, Kaidi Qiu, Qin Xiong, Huiting Lin, Jun Zhang, Haibo Tan

**Affiliations:** ^1^National Navel Orange Engineering Research Center, Gannan Normal University, Ganzhou, China; ^2^Key Laboratory of South China Agricultural Plant Molecular Analysis and Genetic Improvement, Guangdong Provincial Key Laboratory of Applied Botany, South China Botanical Garden, Chinese Academy of Sciences, Guangzhou, China; ^3^Xiangya School of Pharmaceutical Sciences, Central South University, Changsha, China

**Keywords:** endophytic fungus, Gannan navel orange, antibacterial activity, fungal diversity, secondary metabolites

## Abstract

Gannan navel orange is a famous brand in China but the isolation of its endophytic fungi was rarely reported. In this study, a total of 54 strains of endophytic fungi were successfully isolated from the pulp, peel, twig, and leaf of Gannan navel orange; they were successfully identified to belong to 17 species of 12 genera. All these strains were fermented using potato-dextrose agar (PDA) medium, and their secondary metabolites were then extracted with ethyl acetate (EtOAc). The antibacterial assays of *Escherichia coli* (*E. coli*), methicillin-resistant *Staphylococcus aureus* (MRSA), and *Xanthomonas citri* subsp. *citri* (*Xcc*) were also performed for the EtOAc extracts of these strains. As a result, the extracts of both *Geotrichum* sp. (gc-1-127-30) and *Diaporthe biconispora* (gc-1-128-79) demonstrated significant antibacterial activities against *Xcc*, and the MIC value for the extract of *Colletotrichum gloeosporioides* against MRSA was low to 62.5 μg/mL. Moreover, the chemical components of the extracts of *Colletotrichum* sp., *Diaporthe biconispora*, and *Annulohypoxylon atroroseum* were primarily investigated, and they successfully led to the isolation of 24 compounds involving a new botryane sesquiterpene. Among the isolated products, compound **2** showed significant inhibitory activities toward SA, MRSA, *E. coli*, and *Xcc* with MIC values of 12.5, 3.1, 125, and 12.5 μg/mL, respectively. This study revealed that the endophytic fungi of Gannan navel orange showed high potency to produce secondary metabolites with significant antibacterial effects.

## 1. Introduction

China is one of the most important countries for oranges production with an amount of 7.6 million tons in 2022 (USDA, 2022). Gannan, located in the south of Jiangxi province, is a critical cultivation area for orange in China. The Newhall navel orange (*Citrus sinensis* Osbeck cv. Newhall) produced in Gannan, namely Gannan navel orange, is honored as a national geographic indication product in China (Chen et al., 2009; Zhang et al., 2022b).

Plant endophytes have been disclosed to be an extremely important resource for the discovery of new microorganisms (Cabral et al., 1999). In recent years, numerous studies had disclosed that a huge number of endophytic fungi were present in plants, constructing a mutually beneficial symbiotic relationship with their hosts (Collinge et al., 2022). Moreover, most of them showed the capability to help the host to tolerate the external environments (Manzur et al., 2022), promote the growth of plants (Abdalla and Matasyoh, 2014), and biosynthesize some bioactive ingredients to protect the host plants (Mattoo and Nonzom, 2021). Although a large number of studies have been conducted to illustrate the interaction between endophytes and host plants, systematic investigations on the Gannan navel orange and its endophytes are still rare.

Bacterial infection is becoming to be one of the most intractable infectious diseases because of its emerging resistance to the existing antibiotics. Nowadays, the pathogenic bacterium resistant to widely used antibiotics is considered as one of the most serious threats to global public health, which is responsible for serious infections due to high rates of morbidity and mortality in clinical treatment. Among the frequently encountered bacteria, pathogenic *Escherichia coli* usually causes severe diarrhea worldwide. There are nearly 1.7 billion cases of diarrheal disease reported every year (Yang S. C. et al., 2017), and 9.4% of them are caused by diarrheagenic *E. coli* (Indhuprakash et al., 2021), which has resulted in about 760,000 death of children every year from 2011 to 2015 (Chowdhury et al., 2015). Methicillin-resistant *Staphylococcus aureus* (MRSA) is well known as a predominant nosocomial pathogenic bacterium, which represents a crucial crisis in public health systems and poses a serious threat to human life, especially in developed countries (Rebecca, 2008). Therefore, the discovery of novel therapeutic agents that are effective against pathogenic bacteria is of great interest to both medicinal scientists and the pharmaceutical industry to maintain public health in future.

The endophytic fungus is now serving as a promisingly strategic natural bioresource for the discovery of novel natural products with interesting chemical structures and versatile biological activities (Fan et al., 2022). In particular, it was well-respected as a treasure house for developing novel antibacterial agents (Cruz et al., 2020). However, the endophytic fungus of Gannan navel oranges has been rarely investigated. In one of our previous studies, we had isolated and identified an endophytic fungus *Aspergillus aculeatus* GC-09 from Gannan navel orange, the antibacterial screening disclosed that its crude EtOAc extract showed significant antifungal activity against *Penicillium italicum* with a MIC of 31.3 μg/mL, which was comparable to the positive control prochloraz (Zhang et al., 2022a). Except that, there were no reports on the endophytic fungi of Gannan navel oranges.

In order to recognize the abundance and distribution of endophytic fungus species of Gannan navel oranges and illustrate their antibacterial potential as a particular interest, a comprehensive investigation on endophytic fungi from Gannan navel orange was performed in the present study. As a result, 54 strains of endophytic fungi were successfully isolated from the healthy pulp, peel, twig, and leaf of Newhall navel orange for the first time. The molecular identification through the ITS rDNA sequences of the isolated endophytic fungi successfully distinguished them to be 17 species of 12 genera. Moreover, the antimicrobial screening against MRSA, *E. coli*, and *X. citri* subsp. *citri* (*Xcc*) was carried out, revealing that a few fermentation extracts of the isolated endophytic fungi showed significant antibacterial activity. To the best of our knowledge, this study is the first systematic report on the biodiversity, phylogeny, and antibacterial activity of endophytic fungi associated with Gannan navel orange.

## 2. Materials and methods

### 2.1. Plant material and pathogens

Healthy and symptomless Gannan navel oranges (*Citrus sinensis* Osbeck cv. Newhall) were collected from Gannan Normal University Navel Orange Resource Nursery, Ganzhou County, Jiangxi Province, China, in May 2020. *E. coli* (ATCC 12435), SA (CMCC 26003), and MRSA (JCSC 3063) were obtained from Guangdong Microbiology Culture Center (Guangzhou, China), and *X. citri* subsp. *citri* was obtained from the China-USA Citrus Huanglongbing Joint Laboratory.

### 2.2. Isolation and purification of fungal endophytes

The isolation of endophytic fungi from collected plant parts was according to a standard procedure established previously (Li et al., 2020). The pulp, peel, twig, and leaf of Gannan navel orange were used to isolate the corresponding endophytic fungi. The samples were washed with sterile water and dried with sterile filter paper. Then, the cleared tissues were cut into small pieces using a sterile blade and surface-sterilized by sequential immersion in 75% ethanol for 1 min, 1.3 mol/L sodium hypochlorite (3–5% available chlorine) for 30 s, and they were rinsed three times with sterile water between each step simultaneously. These tissue pieces were placed on potato dextrose agar (PDA) plates with 0.4 μg/mL ampicillin and kanamycin to isolate the fungal endophytes. The tissue pieces were observed for mycelia growth. Morphologically distinct isolates were similarly sub-cultured several times to purify and obtain pure isolates. As soon as the fungal mycelium grows out of the tissue slice, the mycelium is transferred to fresh PDA plates for purification and culture. The pure isolates were numbered and kept in a storage tube at −20°C for further study.

### 2.3. ITS sequence processing and molecular identification

The identification of endophytic fungi was based on molecular and morphological analysis. The molecular identification was carried out using the internal transcript spacer regions (ITS1 and ITS4) and the intervening 5.8S rRNA region sequencing. Polymerase chain reaction (PCR) was performed to amplify the ITS region of the fungal isolates using the universal ITS primers, ITS1 (5′-TCCGTAGGTGAACCTGCGG-3′) and ITS4 (5′-TCCTCCGCTTATTGATATGC-3′). The PCR reaction mixture (20 μL) contained 1 μL template (100 ng/μL purified DNA sample), 10 μM of each primer, 0.5 μL 2 × Taq PCR Master Mix, and 8 μL ddH_2_O. PCR conditions performed were as follows: initial denaturation at 95°C for 3 min, followed by 35 cycles of 94°C for 40 s, 52°C for 50 s, and 72°C for 1 min as well as a final extension at 72°C for 10 mins. The amplified PCR products were checked on 1% agarose gel and then sent to Sangon Biotech (Shanghai) Co., Ltd for sequencing. All obtained fungal ITS sequences have been deposited in GenBank and analyzed using BLAST search in the National Center of Biotechnology Information (NCBI) database to compare the sequence homology with closely related organisms.

The ITS sequences were aligned to determine the identity of the corresponding fungal ITS sequences by using BLAST to search from the GenBank database. The endophytic fungus was identified by the percentage identity, and the species level of the targeted endophytic fungus was successfully distinguished with a sequencing identity of more than 99%, whereas the genus level was assigned if sequence identity was between 95 and 99%. Notably, when the sequence identity was determined ≤ 95%, the related endophytic fungus could be only identified at the family or ordinal level by referring to the suggestion of Landeweert et al. (2003).

The sequencing results were analyzed in the SeqMan module of DNAStar 5.0 software for peak plot analysis (Burland, 1999), the sequence fragments were proofread and saved in fasta format and compared with known sequences in Gene bank using BioEdit v7.1 (Hall, 1999, 2011). Then, the sequences were cut and edited, and a matrix was constructed based on the aforementioned results. The sequence output was saved in fas format for phylogenetic tree construction. Bayesian inference was performed by MrBayes v3.1.2 (Ronquist and Huelsenbeck, 2003) with the Markov Chain Monte Carlo algorithm and treated the random initial trees as BI settings. Four chains were run, generating 1,000,000 generations per chain. Sampling was conducted every 1,000 generations, and the first 2,500 trees were discarded as burn-in. The remaining trees were used to construct the majority-rule consensus trees. ClustalW Multiple was initially used to perform alignments by BioEdit. Bootstrap (Felsenstein, 1985) values of internal nodes were obtained with 1,000 replicates. Bayesian inference (BI) was operated with the best-fit model (K80+ G) which was selected based on the AIC criterion using MrModeltest 2.0. *Calophoma aquilegiicola, Didymella glomerata, Leptosphaeria microscopica*, and *Paraboeremia litseae* were selected as outgroup species.

### 2.4. Preparation of EtOAc extracts of different endophytic fungi

Submerged cultivation of the endophytic fungi was carried out to produce secondary metabolites. The strain of the agar with the fungal colony was added to a conical flask (500 mL) with 250 mL potato dextrose broth (PDB) at 28°C for 7 days under constant shaking (150 rpm). After cultivation, the secondary metabolites of each endophyte were extracted by ultrasonic with EtOAc.

### 2.5. Isolation of secondary metabolites

The endophytic fungi *Colletotrichum* sp., *Diaporthe biconispora*, and *Annulohypoxylon atroroseum* were cultured on an autoclaved rice solid medium (5 × 3 L Erlenmeyer flasks, each containing 300 g of grains and 360 mL of distilled water) for 30 days at 28°C. After cultivation, the mycelia and rice solid medium were extracted three times with EtOAc crude extract were obtained. We obtained the main compounds of these two strains that using silica gel chromatography, reversed-phase C_18_ column chromatography, and Sephadex LH-20 column chromatography were isolated.

### 2.6. Antibacterial activity

The EtOAc extracts of each endophytic fungi were evaluated for their antibacterial activity. MIC values were determined by the methodology of micro broth dilution in Mueller-Hinton broth (MHB) medium according to CLSI guidelines, the positive control was kanamycin, vancomycin, and CuSO_4_. Briefly, 180 μL bacteriological solution (1.25 × 10^6^ CFU/mL) with resazurin as an indicator was added into each well of the first row in the 96-well plate, whereas 100 μL bacteriological solution (1.25 × 10^6^ CFU/mL) with resazurin as indicator was added to the other wells in the 96-well plate. A total of 20 μL tested extracts in DMSO solution with a concentration of 10 mg/mL were added to 180 μL bacterial liquid. Then, the method of double dilution was adopted in the 96-well plates. The change of the indicator in the 96-well plates was observed, and the lowest concentration of the drug preventing visible growth of the pathogen was taken as the MIC value (Wang et al., 2022). Similarly, the monomer compounds were also tested.

## 3. Results

### 3.1. Diversity of cultural endophytic fungi from Gannan navel orange

In this study, 54 strains of endophytic fungi were successfully isolated from different tissues of Gannan navel orange, which included the pulp, peel, twig, and leaf. Among these endophytic fungi strains, 2 strains (3.70%) were isolated from pulp, 44 strains (81.48%) were purified from the peel, 5 strains (9.26%) originated from twig, and 3 strains (9.26%) were isolated from leaf (Supplementary Table S1). The results indicated that there were abundant endophytic fungi presenting in the Gannan navel orange. Moreover, this study also suggested that the peel of Gannan navel orange might be the most suitable host place for the endophytic fungi.

### 3.2. ITS identification of the endophytic fungi of Gannan navel orange

**Figure 1:**
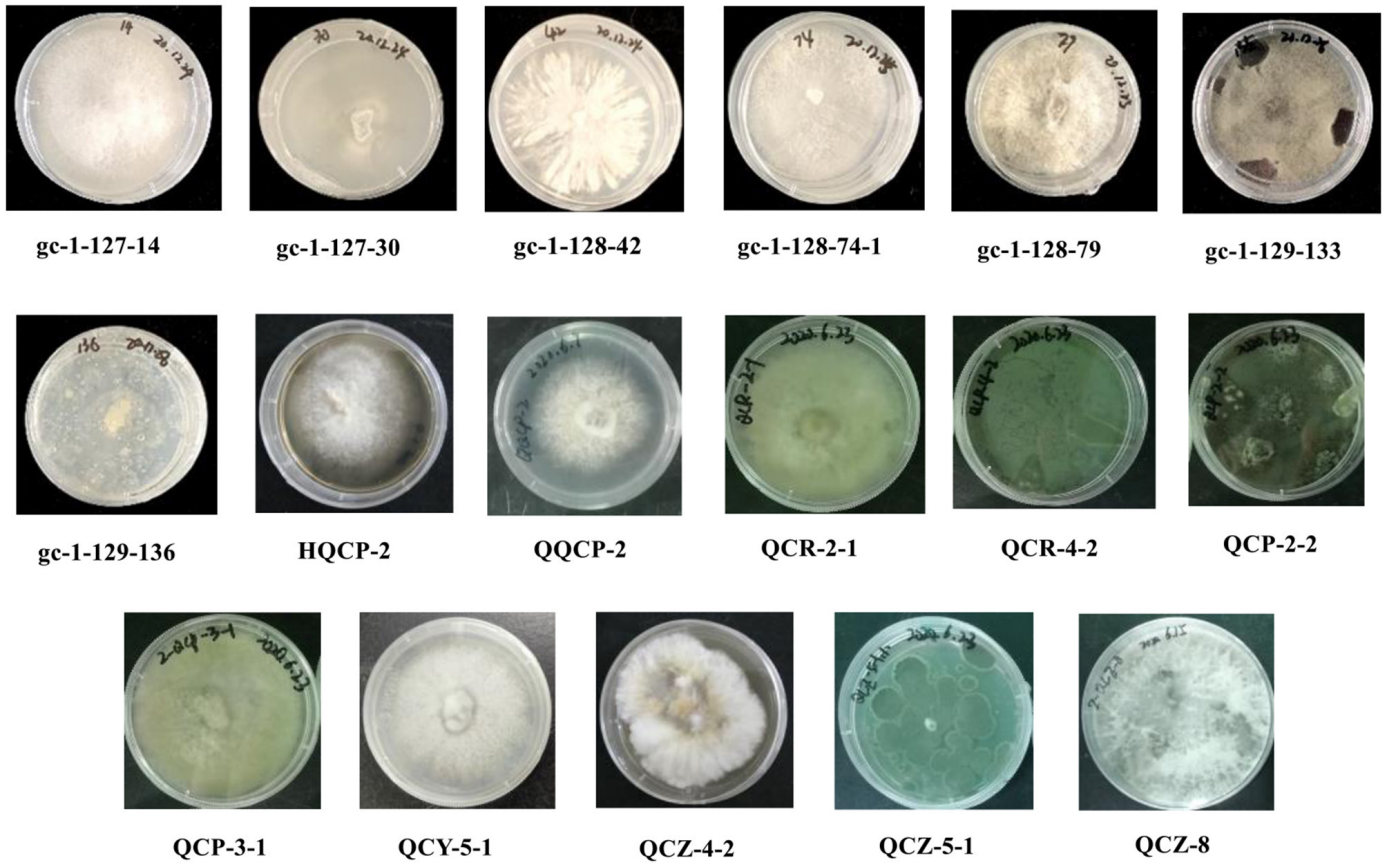
17 strains of endophytic fungi isolated from Gannan navel orange.

**Figure 2:**
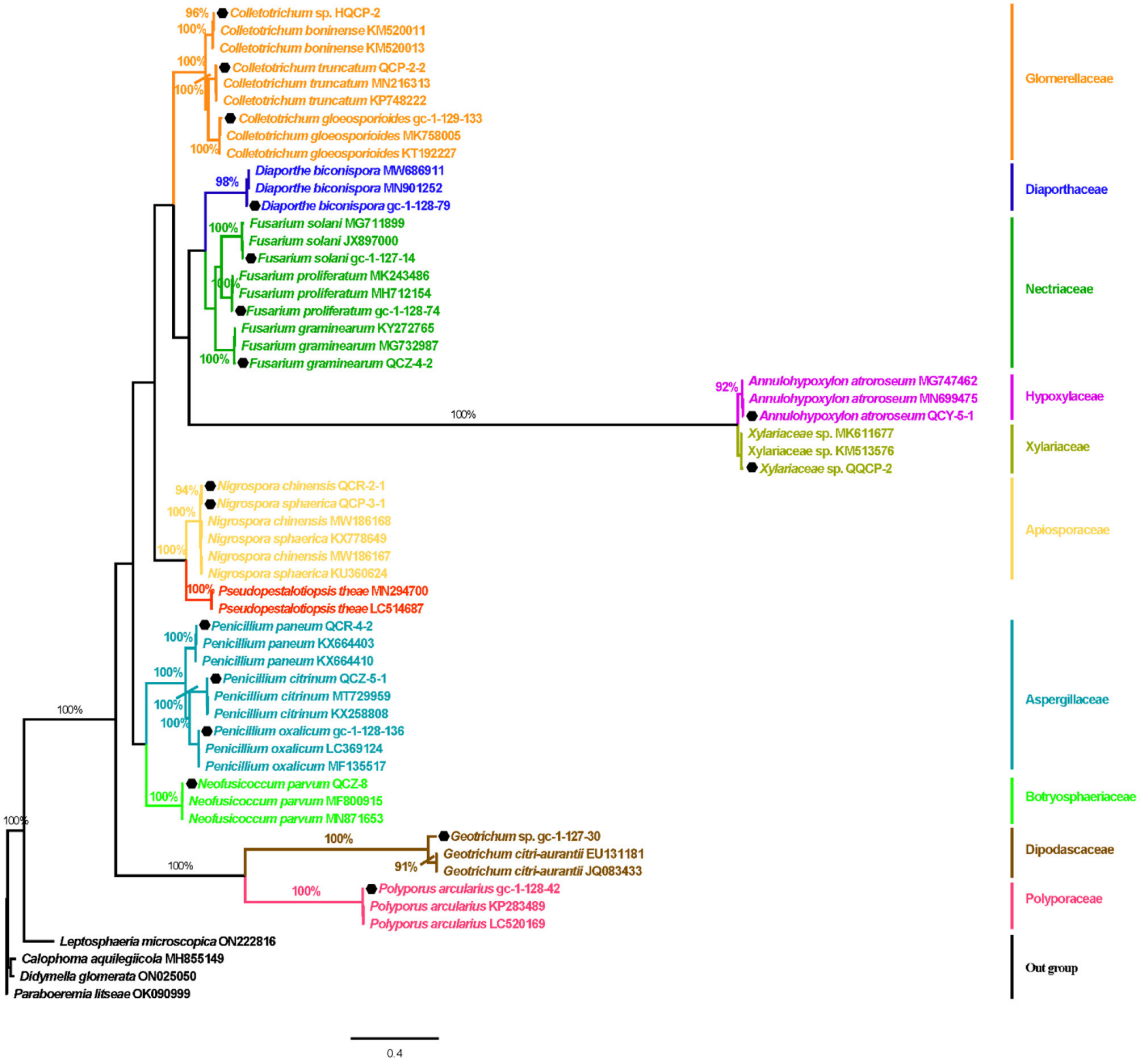
Bayes tree of the Gannan navel orange endophytic fungi based on the ITS sequences. Numbers on branches are support values PP_BI_. PP_BI_ ≥90%, PP_BI_ = 100%. The scale bar represents 0.4 genetic variation per nucleotide sequence. *Leptosphaeria microscopica* (ON222816), *Calophoma aquilegiicola* (MH855149), *Didymella glomerata* (ON025050), and *Paraboeremia litseae* (OK090999) were used as outgroup species.

Based on the inspection and comparison of the fungal morphological characteristics and phenotypes appearance on the PDA culture medium for the 203 purified strains of endophytic fungi, 54 strains showing different morphological features were selected for the molecular biology identification *via* the sequence analysis of the rDNA ITS (internal transcribed spacer) region. From the results, all of the endophytic fungi strains isolated from Gannan navel orange could be assigned to 17 representative morphotypes, as shown in Figure 1. Subsequently, ITS rDNA sequences of each morphotype were generated and submitted to the NCBI database to obtain the corresponding sequence numbers (Table 1). Moreover, a phylogenetic tree of the endophytic fungi of Gannan navel orange was successfully constructed as depicted in Figure 2.

**Table 1:** ITS identification results of endophytes of *Citrus sinensis* Osbeck cv. Newhall.

**Strain No**.	**Homolog sequences**	**Host**	**GenBank No**.	**Closest No**.	**Sequence identity, %**
gc-1-127-14	*Fusarium solani*	Peel	OP238463	JX897000.1	100.00%
gc-1-127-30	*Geotrichum* sp.	Peel	OP238464	OK094899.1	97.84%
gc-1-128-42	*Polyporus arcularius*	Peel	OP238465	JQ283965.1	99.69%
gc-1-128-74-1	*Fusarium proliferatum*	Peel	OP238466	MK243486.1	99.81%
gc-1-128-79	*Diaporthe biconispora*	Peel	OP238467	MN901252.1	99.25%
gc-1-129-133	*Colletotrichum gloeosporioides*	Peel	OP238468	MK758005.1	99.81%
gc-1-129-136	*Penicillium oxalicum*	Peel	OP623448	MW077100.1	99.50%
HQCP-2	*Colletotrichum* sp.	Peel	OP643826	KM520011.1	99.29%
QQCP-2	*Xylariaceae* sp.	Peel	OP643827	KM513576.1	98.59%
QCR-2-1	*Nigrospora chinensis*	Pulp	OP643828	MK834674.1	99.60%
QCR-4-2	*Penicillium paneum*	Pulp	OP643829	KX664403.1	99.27%
QCP-2-2	*Colletotrichum truncatum*	Peel	OP643830	MN216313.1	99.63%
QCP-3-1	*Nigrospora sphaerica*	Peel	OP643831	KX778649.1	99.22%
QCY-5-1	*Annulohypoxylon atroroseum*	Leaf	OP643832	MN699475.1	99.78%
QCZ-4-2	*Fusarium graminearum*	Twig	OP643833	MG732987.1	99.40%
QCZ-5-1	*Penicillium citrinum*	Twig	OP643834	MT729959.1	99.62%
QCZ-8	*Neofusicoccum parvum*	Twig	OP643835	MF800915.1	99.63%

Moreover, the GenBank accession numbers, homolog sequences, sequence identities, and closest accession numbers of the identified endophytic fungal isolates were systematically cataloged and shown in Table 1. Therefore, according to the results of morphological and molecular identification, 17 representative morphotypes were successfully identified belonging to 12 genera. Moreover, it is worth noting that 14 endophytic fungi from the 17 ones were identified as species (Figure 1). Furthermore, all the isolated endophytic fungi were identified to be 2 phyla (Ascomycota and Basidiomycota), 5 classes (*Sordariomycetes, Saccharomycetes, Agaricomycetes, Eurotiomycetes*, and *Dothideomycetes*), 9 orders (*Hypocreales, Saccharomycetales, Polyporales, Diaporthales, Glomerellales, Eurotiales, Glomerellales, Xylariales*, and *Botryosphaeriales*), 10 families (*Nectriaceae, Dipodascaceae, Polyporaceae, Diaporthaceae, Glomerellaceae, Aspergillaceae, Xylariaceae, Apiosporaceae, Hypoxylaceae*, and *Botryosphaeriaceae*), 12 genera, and 17 species in total.

### 3.3. Antibacterial screening of the EtOAc extracts from endophytic fungi cultures

With the fermentation products of the endophytic fungi from Gannan navel orange in hand, the antimicrobial screening for the discovery of potential strain-producing biologically interesting secondary metabolites was then conducted. The antimicrobial results of the tested extracts are depicted in Table 2. The results revealed that the EtOAc extracts of 8 endophytic fungi exhibited obvious inhibitory activities against Gram-negative bacterium *E. coil*, whereas the other 9 EtOAc extracts showed no noticeable antimicrobial activities. Notably, the aforementioned 8 antibacterial strains belonged to 7 genera of *Fusarium, Geotrichum, Fusarium, Diaporthe, Colletotrichum, Penicillium, Annulohypoxylon*, and *Neofusicoccum*.

**Table 2:** Antibacterial activity of endophytic fungi fermented extracts from Gannan navel orange.

**Strain No**.	**Homolog sequences**	**Fermentation yield (mg/L)**	**MIC (**μ**g/mL)**		
			***E. coli***	**MRSA**	***Xcc***
gc-1-127-14	*Fusarium solani*	22.8	500	500	250
gc-1-127-30	*Geotrichum* sp.	37.2	1,000	>1,000	62.5
gc-1-128-42	*Polyporus arcularius*	40.0	>1,000	>1,000	125
gc-1-128-74-1	*Fusarium proliferatum*	105.6	125	500	>1,000
gc-1-128-79	*Diaporthe biconispora*	71.0	500	1,000	31.3
gc-1-129-133	*Colletotrichum gloeosporioides*	47.6	1,000	62.5	250
gc-1-129-136	*Penicillium oxalicum*	126.6	>1,000	>1,000	1,000
HQCP-2	*Colletotrichum* sp.	421.6	500	250	500
QQCP-2	*Xylariaceae* sp.	1,158.8	>1,000	>1,000	>1,000
QCR-2-1	*Nigrospora chinensis*	466.0	>1,000	>1,000	>1,000
QCR-4-2	*Penicillium paneum*	2,445.6	250	>1,000	1,000
QCP-2-2	*Colletotrichum truncatum*	1,765.2	>1,000	>1,000	>1,000
QCP-3-1	*Nigrospora sphaerica*	440.8	>1,000	>1,000	>1,000
QCY-5-1	*Annulohypoxylon atroroseum*	14,211.2	1,000	>1,000	>1,000
QCZ-4-2	*Fusarium graminearum*	524.4	>1,000	1,000	>1,000
QCZ-5-1	*Penicillium citrinum*	2,644.4	>1,000	>1,000	>1,000
QCZ-8	*Neofusicoccum parvum*	361.2	1,000	>1,000	>1,000
Kanamycin			0.63	/	/
Vancomycin			/	1.25	/
CuSO_4_			/	/	125

Moreover, the antibacterial activities against the Gram-positive bacterium MRSA and plant pathogenic bacterial *Xcc* were also conducted for the 17 EtOAc extracts of the isolated endophytic fungi. As a result, 6 EtOAc extracts of endophytic fungi exhibited significant inhibitory activities against MRSA, involving *Fusarium solani, Fusarium proliferatum, Diaporthe biconispora, Colletotrichum gloeosporioides, Colletotrichum* sp., and *Fusarium graminearum*. Notably, among them, *Colletotrichum gloeosporioides* (gc-1-129-133) demonstrated the highest antibacterial activity with a MIC value of 62.5 μg/mL, which was closely comparable to that of the clinical drug vancomycin (positive control, MIC = 1.3 μg/mL), showing significant potential for the discovery of novel antibacterial drugs. Similarly, some EtOAc extracts of the endophytic fungi exhibited considerable inhibitory activities against the plant pathogenic fungus *Xcc*, which were *Fusarium solani, Geotrichum* sp., *Polyporus arcularius, Diaporthe biconispora, Colletotrichum gloeosporioides, Penicillium oxalicum, Colletotrichum* sp., and *Penicillium paneum*. Interestingly, both the EtOAc extracts of *Geotrichum* sp. (gc-1-127-30) and *Diaporthe biconispora* (gc-1-128-79) demonstrated very potent antibacterial activity against *Xcc* with MIC values as low as 62.5 and 31.3 μg/mL, respectively, strongly suggesting that these two endophytic fungi might be severed as promising natural bioresources for the discovery of novel antibacterial agents.

Taken together, the endophytic fungus *Fusarium solani* showed the best antibacterial activities against all of the tested bacteria involving *E. coli* (MIC = 500 μg/mL), MRSA (MIC = 500 μg/mL), and *Xcc* (MIC = 250 μg/mL), which were much more potent than other sixteen endophytic fungi from Gannan navel orange (Table 2). Notably, the attractable results also came from the endophytic fungus *Fusarium solani*, which showed comprehensive antibacterial activities against all of the Gram-negative bacterium (*E. coli*), Gram-positive bacterium (MRSA), and pathogenic fungus (*Xcc*), suggesting that the EtOAc extract of the endophytic fungus *Fusarium solani* might be developed as promising biocontrol agent with broad-spectrum antibacterial effect.

### 3.4. Isolation of the EtOAc extracts

Based on the antibacterial effects of all EtOAc extracts tested, three endophytic fungi *Colletotrichum* sp., *D. biconispora*, and *A. atroroseum* were selected for the discovery of antibacterial novel secondary metabolites by column chromatography method.

**Figure 3:**
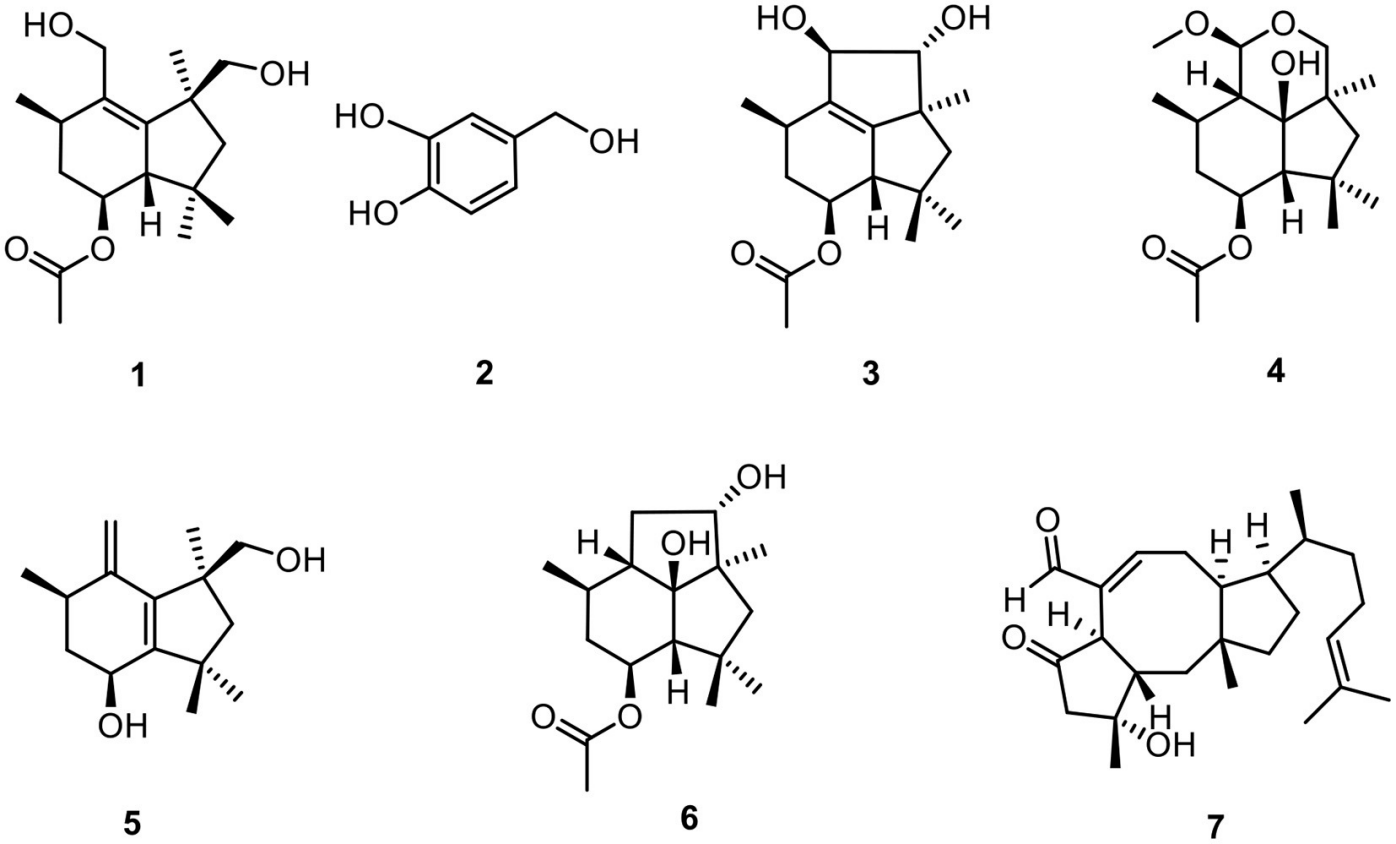
The structures of compounds **1**–**7**.

As a result, a new botryane sesquiterpene colletene A (**1**) and six known compounds 3,4-dihydroxybenzyl alcohol (**2**) (Du et al., 2011), 4β-acetoxy-9β,l0β,15α-trihydroxyprobotrydial (**3**) (Collado et al., 1995), *O*-methyldihydrobotrydial (**4**) (Kimura et al., 1988), botry-[1(10),5(9)-^2^*H*]-dien-[15-^2^*H*]-ol (**5**) (Daoubi et al., 2006), ergosta-4,6,8(14),22-tetraen-3-one (**6**) (Durán-Patrón et al., 2001), and 4β-acetoxyprobotryane-9β,15α-diol (**7**) (Wei et al., 2004) were successfully isolated from *Colletotrichum* sp. (Figure 3).

Colletene A (**1**) was obtained as a colorless oil with the chemical molecular formula of C_17_H_28_O_4_, which was successfully established on the basis of (+)-HRESIMS *m/z* 319.1887 [M + Na]^+^ (calcd for C_17_H_28_NaO_4_, 319.1880), implying four degrees of hydrogen deficiency. The ^1^H NMR data (Table 3) showed typical signals for five methyl groups [δ_H_ 0.88 (3H, s, H-12), 1.05 (3H, s, H-13), 1.15 (3H, d, H-11), 1.39 (3H, s, H-14), and 2.04 (3H, s, H-17)]. The ^13^C NMR and HSQC spectra of **1** revealed the presence of 17 carbon resonances, including five methyls (δ_C_ 20.5, 21.6, 23.7, 24.5, and 29.6), four methylenes involving two oxygen carbons (δ_C_ 38.6, 55.1, 59.0, and 71.2), three methines involving an oxygen carbon (δ_C_ 35.1, 57.0, and 71.7), and five non-protonated carbons (two quaternary carbons at δ_C_ 38.7 and 46.6, two olefinic carbons at δ_C_ 135.7 and 144.8, a carbonyl carbon at δ_C_ 170.7). These results strongly suggested that compound **1** might be a sesquiterpene derivative.

**Table 3:** ^1^H (500 MHz) and ^13^C (125 MHz) NMR data of **1** in CDCl_3_.

**1**		
**No**.	δ_H_ **(*****J*** **in Hz)**	δ_C_
1		135.7, C
2	2.53, qt (6.8, 10.6)	35.1, CH
3a	1.34, overlapped	38.6, CH_2_
3b	2.07, m	
4	4.87, m	71.7, CH
5	2.36, dd (3.4, 9.7)	57.0, CH
6		38.7, C
7α	1.32, overlapped	55.1, CH_2_
7β	1.59, d (13.0)	
8		46.6, C
9		144.8, C
10a	4.00, d (11.8)	59.0, CH_2_
10b	4.31 d (11.8)	
11	1.15 d (6.8)	20.5, CH_3_
12	1.05, s	29.6, CH_3_
13	0.88, s	23.7, CH_3_
14	1.39, s	24.5, CH_3_
15	3.38, m	71.2, CH_2_
16		170.7, C
17	2.04, s	21.6, CH_3_

**Figure 4:**
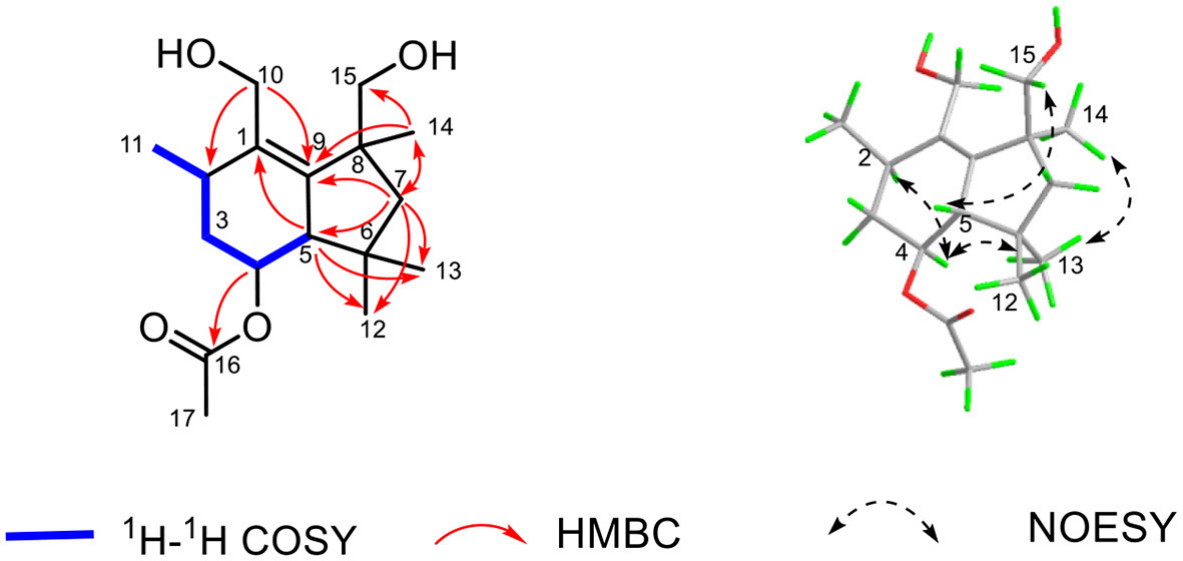
Key HMBC, ^1^H-^1^H COZY, and NOESY correlations of **1**.

The established functional groups chemo-logically accounted for two degrees of hydrogen deficiency. Therefore, the remaining two degrees of hydrogen deficiency necessitated that compound **1** should possess a bicyclic ring system. In order to construct the bicyclic skeleton of compound **1**, the 2D NMR spectra involving both the HMBC and ^1^H-^1^H COSY (Figure 4) spectra were performed and elucidated. First, the ^1^H-^1^H COSY correlations of H_3_-11/H-2/H_2_-3/H-4/H-5 informatively suggested the presence of one independent spin system. The key HMBC correlations from H_2_-10 to C-2 and C-9, H_3_-14 to C-7, C-9, and C-15, H-5 to C-1, C-12, and C-13, H_2_-7 to C-5, C-9, C-12, C-13, C-14, and C-15 could readily conclude the existence of rings A-B, which formatted as a 6/5 fused bicyclic ring system. The aforementioned result also coincides with its hydrogen deficiency. Moreover, the obvious HMBC correlation from H-4 to C-16 further confirmed that the carbonyl group should be attached at the C-4 position. Therefore, the planar structure of **1** was completely established, which suggested that compound **1** should be a new botryane sesquiterpene.

The relative configuration of **1** was determined by the careful analysis of its NOESY spectrum (Figure 4). The key NOESY signals between H-4 to H-2 and H_3_-13 as well as H_3_-13 to H_3_-14 informatively suggested that these protons were on the same side and assigned as α-orientation. Then, the NOESY correlations of H-5 to H_2_-15 demonstrated that these protons were β-orientated. Notably, the absolute configuration of **1** could not be determined because of its weak Cotton effects. Therefore, the chemical structure of **1** was finally established as depicted in Figure 4, and named as colletene A.

**Figure 5:**
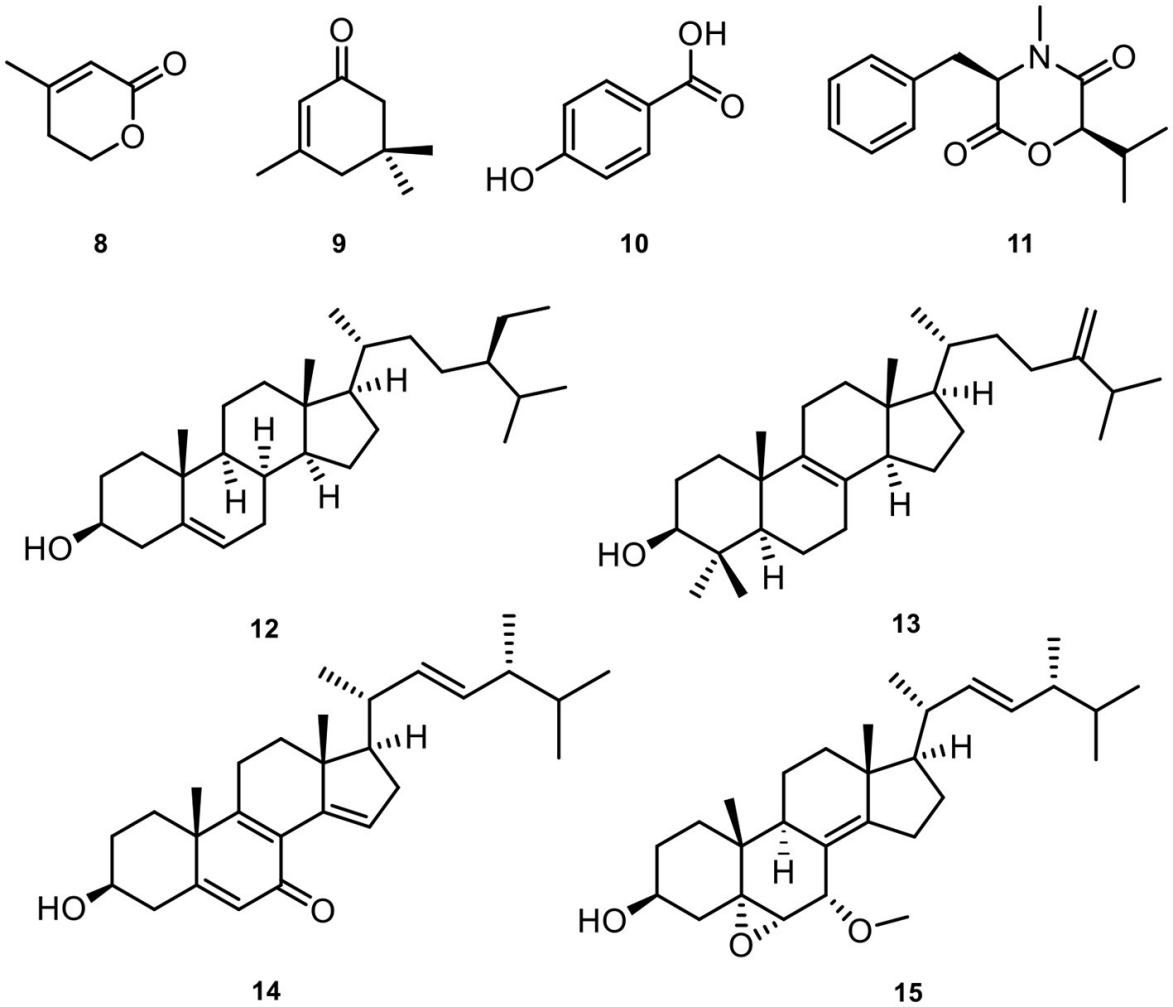
The structures of compounds **8**–**15**.

Meanwhile, eight known compounds were obtained from *Diaporthe biconispora*, including 3-methyl-2-penten-5-olide (**8**) (Shimomura et al., 2008), isophorone (**9**) (Tarantilis and Polissiou, 1997), *p*-hydroxy benzoic acid (**10**) (Boonyaketgoson et al., 2015), lateritin (**11**) (Hasumi et al., 1993), β-sitosterol (**12**) (Prakash and Chaturvedula, 2012), 4,4-dimethyl-5α-ergosta-8,24(28)-dien-3β-ol (**13**) (Fattorusso et al., 1992), 3β-hydroxyl-(22*E*, 24*R*)-ergosta-5,8,14,22-tetraen-7-one (**14**) (Wang et al., 2008), 22*E*-7α-methoxy-5α,6α-epoxyergosta-8(14),22-dien-3β-ol (**15**) (Gao et al., 2010). The chemical structures of the isolated natural products from *D. biconispora* are given in Figure 5.

**Figure 6:**
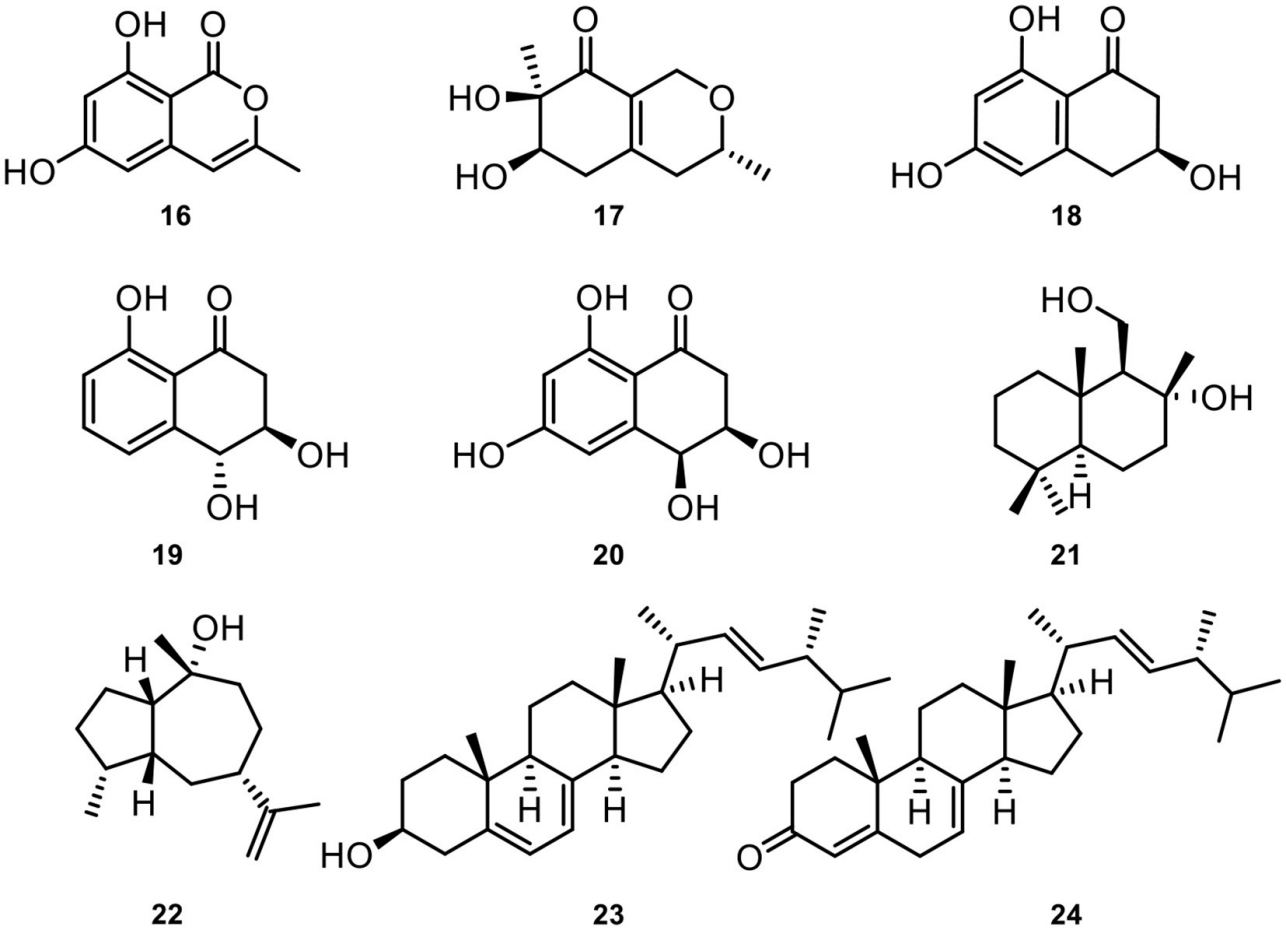
The structures of compounds **16**–**24**.

In addition, nine known compounds involving 6,8-dihydroxy-3-methylisocoumarin (**16**) (Feng et al., 2020), xylariphilone (**17**) (Arunpanichlert et al., 2016), scytalone (**18**) (Yang et al., 2020), *trans*-3,4-dihydro-3,4,8-trihydroxynaphthalen-1(2*H*)-one (**19**) (Couché et al., 2009), *cis*-4-hydroxyscytalone (**20)** (Couché et al., 2009), 8-hydroxyl-drimanol (**21**) (Li et al., 2018), pogostol (**22**) (Amand et al., 2012), ergosterol (**23**) (Li et al., 2014), (22*E*,24*R*)-ergosta-4,7,22-trien-3-one (**24**) (Zhang et al., 2009) from *Annulohypoxylon atroroseum*. The chemical structures of these isolated natural products could be seen in Figure 6.

All the isolated compounds were evaluated for antibacterial activities against SA, MRSA, *E. coli*, and *Xcc*. Notably, the phenolic compound **2** obtained from *Colletotrichum* sp. showed significant inhibitory effects toward all the tested bacterial strains of SA, MRSA, *E. coli*, and *Xcc* with MIC values of 12.5, 3.1, 125, and 12.5 μg/mL, respectively (Table 4). Satisfactorily, these results are consistent with our previous screening experiment for the EtOAc crude extract of *Colletotrichum* sp. Moreover, compounds **19** and **22** isolated from *A. atroroseum* also showed weak antibacterial activities against SA, MRSA, and *Xcc*.

**Table 4:** Antibacterial activity of compounds **1–7, 16–24**.

**Compounds**	**MIC (**μ**g/mL)**			
	**SA**	**MRSA**	***E. coli***	***Xcc***
1	>1,000	>1,000	>1,000	>1,000
2	12.5	3.1	125	12.5
3	250	500	1,000	125
4	>1,000	>1,000	>1,000	250
5	>1,000	>1,000	>1,000	125
6	>1,000	>1,000	>1,000	250
7	>1,000	>1,000	>1,000	250
16	500	500	1,000	250
17	>1,000	1,000	1,000	>1,000
18	1,000	1,000	1,000	1,000
19	250	250	1,000	125
20	>1,000	>1,000	>1,000	500
21	1,000	1,000	1,000	500
22	250	250	1,000	125
23	>1,000	>1,000	>1,000	1,000
24	>1,000	>1,000	>1,000	1,000
Vancomycin	0.63	1.25	/	/
Polymyxin B	/	/	2.5	/
CuSO_4_	/	/	/	125

## 4. Discussion

The fruits of Gannan navel orange contain abundant biologically meaningful chemical components with significant health benefits such as flavonoids (Tripoli et al., 2007), polyphenols (Hu et al., 2021), vitamins (Liu et al., 2010), essential oils (Bermejo et al., 2011), carotenoids (Yang C. et al., 2017), limonenes (Eldahshan and Halim, 2016), and other beneficial chemicals (Cai and Rui, 2013), which shared significantly pharmaceutical and nutritional bio-functions such as anti-cancer (Raghavan and Gurunathan, 2021), anti-oxidation (Zhu et al., 2018), antibacterial (Kawaguchi et al., 2004), lowering cholesterol level (Chau et al., 2004), and other related biological effects. Moreover, its peel has been evidenced to show very potent antimicrobial and anticancer activities (Yang C. et al., 2017; Guo et al., 2018), and it was widely used as healthy food supplants.

The significant health benefits and potent biological activities of Gannan navel orange suggested that its endophytic fungi might also be interesting. In the present study, a total of 54 endophytic fungi were isolated from Gannan navel orange fruits. Meanwhile, we also tested the antibacterial activity of the EtOAc extracts of most of the isolated endophytic fungi against Gram-negative bacterium *E. coli*, MRSA, and *Xcc* as three representative pathogenic bacteria for the first time. Therefore, this study will provide some valuable information for the wide utility of endophytic fungi of Gannan navel oranges and facilitate the development of the Gannan navel oranges industry as well.

In this study, we have successfully isolated and identified 17 strains of endophytic fungi from the Gannan navel orange (*C. sinensis* Osbeck cv. Newhall). To the best of our knowledge, this is the first comprehensive investigation on the isolation and identification of endophytic fungi from healthy Gannan navel orange tissues. As shown in Table 1, most of the endophytic fungi such as *Fusarium solani* (Kurt et al., 2020), *Geotrichum* sp. (Zhao et al., 2020), *Fusarium proliferatum* (Hyun et al., 2000), *Diaporthe biconispora* (Huang et al., 2015), *Colletotrichum gloeosporioides* (Ramos et al., 2016), *Penicillium oxalicum* (Kaur et al., 2020), *Xylariaceae* sp. (Ho et al., 2012), *Colletotrichum truncatum* (Cheng et al., 2013), *Penicillium citrinum* (Sadeghi et al., 2019), and *Neofusicoccum parvum* (Bezerra et al., 2021) have been previously discovered in the genus of *Citrus* as pathogenic fungi. Notably, the *Polyporus arcularius, Penicillium paneum, Nigrospora chinensis, Nigrospora sphaerica, Annulohypoxylon atroroseum*, and *Fusarium graminearum* were discovered from the family of *Citrus* for the first time, and they might be served as characteristic fungi of Gannan navel orange, although more evidences should be provided to support this statement in future study.

Moreover, this study has not only first investigated the endophytic fungi of Gannan navel orange, but also provided some valuable information for biologists to explore the potent relationship between the endophytic fungi and their host plants. The investigation of its rarely studied endophytic fungi might broaden the microcosmic knowledge and illustrate their pharmaceutical potency. Therefore, this study greatly enriched the species diversity of endophytic fungi of Gannan navel orange, and it laid a good foundation for the subsequent research on the endophytic resource of Gannan navel orange.

Endophytic fungi have been extensively investigated due to their remarkable ability for the production of a variety of secondary metabolites with pronounced pharmaceutical activity and interesting chemical structures (Newman and Cragg, 2020). In addition, endophytic fungi usually play important roles in the protection of host plants and produce different bioactive natural products under different certain conditions (Wang et al., 2023), which makes them much more interesting for natural product discovery and plant-microbial interaction (Schulz et al., 2002). In this study, we tested the inhibitory effect of secondary metabolites of endophytic fungi of navel orange against three different pathogens, and many of them showed considerable antimicrobial effects. The results strongly suggested that the endophytic fungal types of Gannan navel orange are rich and diverse. Moreover, this study also provides scientific data for the exploitation of endophytic fungal resources of Gannan navel orange. Metabolites from endophytic fungi have great potential for the discovery of pharmaceutical agents, and the present study provided important information for the discovery of antibacterial natural compounds from the endophytic fungi of Gannan navel orange.

In particular, many countries have encountered huge economic losses because of the severity of CBC caused by *Xcc*. The traditional treatment of the disease with copper-based chemical sprays and streptomycin has led to the emergence of resistant *Xanthomonas* strains (Martins et al., 2020). The development of alternative biocontrol agents has now become a major challenge for scientists. In the antibacterial activity screening, the crude extracts of *Colletotrichum* sp., *D. biconispora* have better inhibitory effects against *Xcc*. Therefore, we have performed the chemical composition investigation and an anti-*Xcc* assay of individual compounds isolated from *Colletotrichum* sp. and *D. biconispora*. The active compound **2** might be responsible for the antibacterial effects of the EtOAc extracts. The current results suggested that it might be a prospective and promising way to discover anti-*Xcc* pesticides from *Citrus* plants itself based on the protective effect of endophytic fungi on the host plant.

Furthermore, we also selected *A. atroroseum* for the chemical study, which successfully resulted in the isolation of nine compounds involving isocoumarin, azaphilones, tetralones, and steroids, but none of these compounds showed significant antibacterial activity against SA, MRSA, and *E. coli*. More bioassay-guided isolation strategies should be performed in our future study to identify highly potent antibacterial compounds from the isolated endophytes. Moreover, the biological evaluations of Gannan navel orange endophytic fungi in this study were limited by the experimental platforms, other biological activities such as the antiviral, anti-inflammatory, antifungal, and various enzyme inhibitory effects should be carried out to fully illustrate the pharmaceutical potential of endophytic fungi of Gannan navel orange in future.

## 5. Summary

In conclusion, the identification of endophytic fungi from Gannan navel orange, the antibacterial screening of their crude fermentation products, and the isolation of bioactive compounds from the EtOAc extracts of two bioactive endophytic fungi were carried out in the present study. A total of 54 strains belonging to 17 species of 12 genera were successfully isolated from Gannan navel orange for the first time. The EtOAc extract of *Fusarium proliferatum* (gc-1-128-74-1) exhibited excellent antibacterial activity against *E. coli*, and the EtOAc extracts of both *Geotrichum* sp. (gc-1-127-30) and *Diaporthe biconispora* (gc-1-128-79) demonstrated significant antibacterial activity against *Xcc*. In addition, The MIC value of the EtOAc extract of *Colletotrichum gloeosporioides* against MRSA was comparable to the positive control vancomycin. Furthermore, 24 compounds involving a new compound were isolated from the EtOAc extracts of *Colletotrichum* sp., *D. biconispora*, and *A. atroroseum*. In addition, compound **2** showed significant inhibitory activities toward SA, MRSA, *E. coli*, and *Xcc* with MIC values of 12.5, 3.1, 125, and 12.5 μg/mL. The present results indicated that the endophytic fungi of Gannan navel orange were abundant and had great potential to produce antibacterial compounds against *E. coli*, SA, MRSA, and *Xcc* deserving more attention in future studies.

## Data availability statement

The datasets presented in this study can be found in online repositories. The names of the repository/repositories and accession number(s) can be found in the article/Supplementary material.

## Author contributions

HW conducted the experiment and collected the experimental data. ZL guided the molecular experimental and phylogenetic analysis. YC and KQ evaluated the activities of the extracts. FD and QX revised the manuscript. HL performed the experiments of compounds isolation. JZ and HT conceived and designed the experiments. All authors contributed to the article and approved the submitted version.
