# Supplementary material for: Isolation, identification, and antibacterial evaluation of endophytic fungi from Gannan navel orange
Source: Front Microbiol. 2023 Jun 15;14:1172629. doi: 10.3389/fmicb.2023.1172629 (PMC10307966; doi:10.3389/fmicb.2023.1172629)
Supplement: Supplementary file 1 [file Data_Sheet_1.pdf]

Supplementary Material

Isolation, identification, and antibacterial evaluation of endophytic fungi from Gannan navel orange

Huan Wang[1,2,†], Ziyue Liu[1,2,†], Fangfang Duan[2], Yan Chen[2,3], Kaidi Qiu[2], Qin Xiong[1], Huiting Lin[1], Jun Zhang[1,*], Haibo Tan[1,2,*]

[1] National Navel Orange Engineering Research Center, Gannan Normal University, Ganzhou 341000, People's Republic of China

[2] Key Laboratory of South China Agricultural Plant Molecular Analysis and Genetic Improvement, Guangdong Provincial Key Laboratory of Applied Botany, South China Botanical Garden, Chinese Academy of Sciences, Guangzhou 510650, People's Republic of China

[3] Xiangya School of Pharmaceutical Sciences, Central South University, Changsha 410013, People's Republic of China

*** Correspondence:**
Corresponding Author
bri71527152@outlook.com (J. Zhang); tanhaibo@scbg.ac.cn (H.B. Tan).

Contents

Spectral data of the compound 1

Table S1. ITS identification results of 54 endophytes of *Citrus sinensis* Osbeck cv. Newhall.

Figure S1. HRESIMS spectrum of compound **1**.

Figure S2. ^{1}H NMR spectrum (500 MHz, $CDCl_3$) of compound **1**.

Figure S3. ^{13}C NMR spectrum (125 MHz, $CDCl_3$) of compound **1**.

Figure S4. ^{1}H-^{1}H COSY spectrum of compound **1**.

Figure S5. HSQC spectrum of compound **1**.

Figure S6. HMBC spectrum of compound **1**.

Figure S7. NOESY spectrum of compound **1**.

Figure S8. UV spectrum of compound **1**.

Figure S9. IR spectrum of compound **1**.

Spectral data of the compound 1: colorless oil; $[\alpha]_D^{25}$ +3 (*c* 0.1, MeOH); ECD (MeOH) λ_{max} ($\Delta\varepsilon$): 200 (+3.72), 230 (−0.45); UV (MeOH) λ_{max} (log ε): 204 (2.72) nm; IR ν_{max} : 3361, 2927, 1714, 1463, 1396, 1244, 1053, 1024, 962, 532, 507 cm^{-1}, ^{1}H (500 MHz) and ^{13}C (125 MHz) NMR data, see **Table 3**. HRESIMS: *m/z* 319.1887 $[M + Na]^+$ (calcd for $C_{17}H_{28}NaO_4$, 319.1880).

Table S1. ITS identification results of 54 endophytes of *Citrus sinensis* Osbeck cv. Newhall.

Genera	Species groups	Strain No.	Host	Sequence identity, %	Closest number
Annulohypoxylon	*A. atroroseum*	QCY-5-1	Leaf	99.78%	MN699475.1
	Annulohypoxylon sp.	QCY-8-1	Leaf	98.16%	LC496556.1
Colletotrichum	*Colletotrichum*. sp.	HQCP-2	Peel	99.29%	KM520011.1
	C. truncatum	QCP-2-2	Peel	99.63%	MN216313.1
	C. gloeosporioides	gc-1-129-133	Peel	99.81%	MK758005.1
Diaporthe	*D. biconispora*	gc-1-128-79	Peel	99.25%	MN901252.1
Fusarium	*F. graminearum*	QCZ-4-2	Twig	99.40%	MG732987.1
	F. solani	gc-1-127-14	Peel	100.00%	JX897000.1
	F. solani	gc-1-127-19	Peel	99.82%	MH477738.1
	F. solani	gc-1-129-127	Peel	99.81%	JX897000.1
	F. solani	gc-1-129-130	Peel	99.62%	KU377470.1
	F. solani	gc-1-129-138	Peel	99.25%	KU377470.1
	F. solani	gc-1-129-140	Peel	99.44%	KF679356.1
	F. solani	gc-1-130-154	Peel	99.62%	MW600440.1
	F. solani	gc-1-130-155	Peel	99.81%	JX897000.1
	F. solani	gc-1-130-175	Peel	99.62%	KY051566.1
	F. solani	gc-1-130-180	Peel	99.81%	JX897000.1
	F. proliferatum	gc-1-128-74-1	Peel	99.81%	MK243486.1
	F. proliferatum	gc-1-128-81	Peel	99.06%	MH712154.1
	F. proliferatum	gc-1-128-83	Peel	99.61%	MH707087.1
	F. proliferatum	gc-1-128-84	Peel	99.25%	MG543763.1
	F. proliferatum	gc-1-128-85	Peel	99.62%	MH712154.1
	F. proliferatum	gc-1-129-135	Peel	99.29%	MG562501.1
	F. proliferatum	gc-1-129-141	Peel	99.25%	MN955525.1
	F. proliferatum	gc-1-129-142-1	Peel	99.62%	MT476359.1
	F. proliferatum	gc-1-129-143	Peel	99.25%	MH712157.1
	F. proliferatum	gc-1-130-170	Peel	99.61%	MF687282.1
	F. proliferatum	gc-1-130-177	Peel	99.61%	MF687307.1
	F. proliferatum	gc-1-130-178	Peel	99.42%	MG543768.1
	F. proliferatum	gc-1-131-202	Peel	99.81%	MN511330.1
	Fusarium. sp.	HQCP-4	Peel	95.05%	ON527751.1
Geotrichum	*Geotrichum* sp.	gc-1-127-3	Peel	95.53%	OK094899.1
	Geotrichum sp.	gc-1-127-22	Peel	96.76%	JQ425851.1
	Geotrichum sp.	gc-1-127-23	Peel	96.39%	JQ425847.1
	Geotrichum sp.	gc-1-127-26	Peel	96.15%	MH612892.1
	Geotrichum sp.	gc-1-127-30	Peel	97.84%	OK094899.1
	Geotrichum sp.	gc-1-127-32	Peel	95.92%	OW983732.1

Genera	Species groups	Strain No.	Host	Sequence identity, %	Closest number
	Geotrichum sp.	gc-1-127-36	Peel	96.63%	MH612889.1
	Geotrichum sp.	gc-1-127-38	Peel	96.58%	EU131181.1
	Geotrichum sp.	gc-1-127-45	Peel	95.17%	JQ425851.1
	Geotrichum sp.	gc-1-130-161	Peel	95.90%	OK094899.1
	Geotrichum sp.	gc-1-131-203	Peel	95.07%	MH612892.1
Neofusicoccum	*N. parvum*	QCZ-8	Twig	99.63%	MF800915.1
Nigrospora	*N. chinensis*	QCR-2-1	Pulp	99.60%	MK834674.1
	N. chinensis	QCY-2-2	Pulp	99.80%	MK834674.1
	N. sphaerica	QCP-3-1	Peel	99.22%	KX778649.1
Penicillium	*P. oxalicum*	gc-1-129-136	Peel	99.50%	MW077100.1
	P. citrinum	QCZ-5-1	Twig	99.62%	MT729959.1
	Penicillium. sp.	QCZ-5-2	Twig	98.91%	MH884073.1
	P. paneum	QCZ-4-1	Twig	99.28%	KX664403.1
	P. paneum	QCR-4-2	Pulp	99.27%	KX664403.1
Polyporus	*P. arcularius*	gc-1-127-42	Peel	99.69%	JQ283965.1
	P. arcularius	gc-1-128-74-2	Peel	99.19%	LC415540.1
Xylariaceae	*Xylariaceae.* sp.	QQCP-2	Peel	98.59%	KM513576.1

Mass Spectrum SmartFormula Report

Analysis Info			
Analysis Name	D:\Data\MS\data\202305\wanghuan_WH-C-150_pos_11_01_15057.d	Acquisition Date	5/18/2023 10:52:23 AM
Method	LC_Direct Infusion_pos_70-500mz.m	Operator	SCSIO
Sample Name	wanghuan_WH-C-150_pos	Instrument	maXis 255552.00029
Comment			

Acquisition Parameter					
Source Type	ESI	Ion Polarity	Positive	Set Nebulizer	0.4 Bar
Focus	Active	Set Capillary	4500 V	Set Dry Heater	180 °C
Scan Begin	70 m/z	Set End Plate Offset	-500 V	Set Dry Gas	4.0 l/min
Scan End	1500 m/z	Set Charging Voltage	0 V	Set Divert Valve	Waste
		Set Corona	0 nA	Set APCI Heater	0 °C

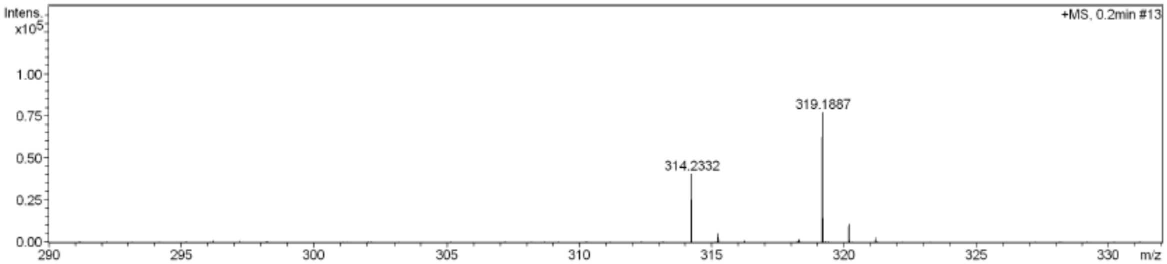

Meas. m/z	#	Ion Formula	Score	m/z	err [ppm]	err [mDa]	mSigma	rdb	e⁻ Conf	N-Rule
314.2332	1	C17H32NO4	100.00	314.2326	2.0	0.6	36.2	2.5	even	ok
319.1887	1	C17H28NaO4	100.00	319.1880	-2.2	-0.7	27.1	3.5	even	ok
593.4059	1	C34H57O8	100.00	593.4048	1.9	1.2	43.8	6.5	even	ok
610.4285	1	C34H60NO8	100.00	610.4313	4.7	2.9	123.1	5.5	even	ok
615.3870	1	C34H56NaO8	100.00	615.3867	0.4	0.3	43.6	6.5	even	ok

Figure S1. HRESIMS spectrum of compound **1**.

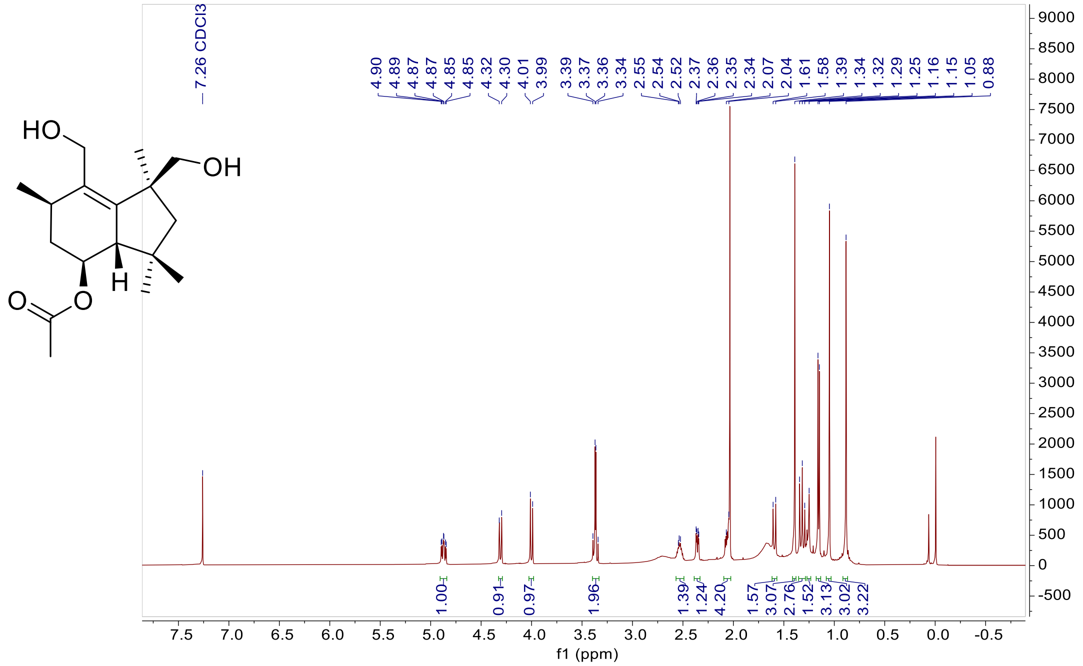

Figure S2. ^{1}H NMR spectrum (500 MHz, $CDCl_3$) of compound **1**.

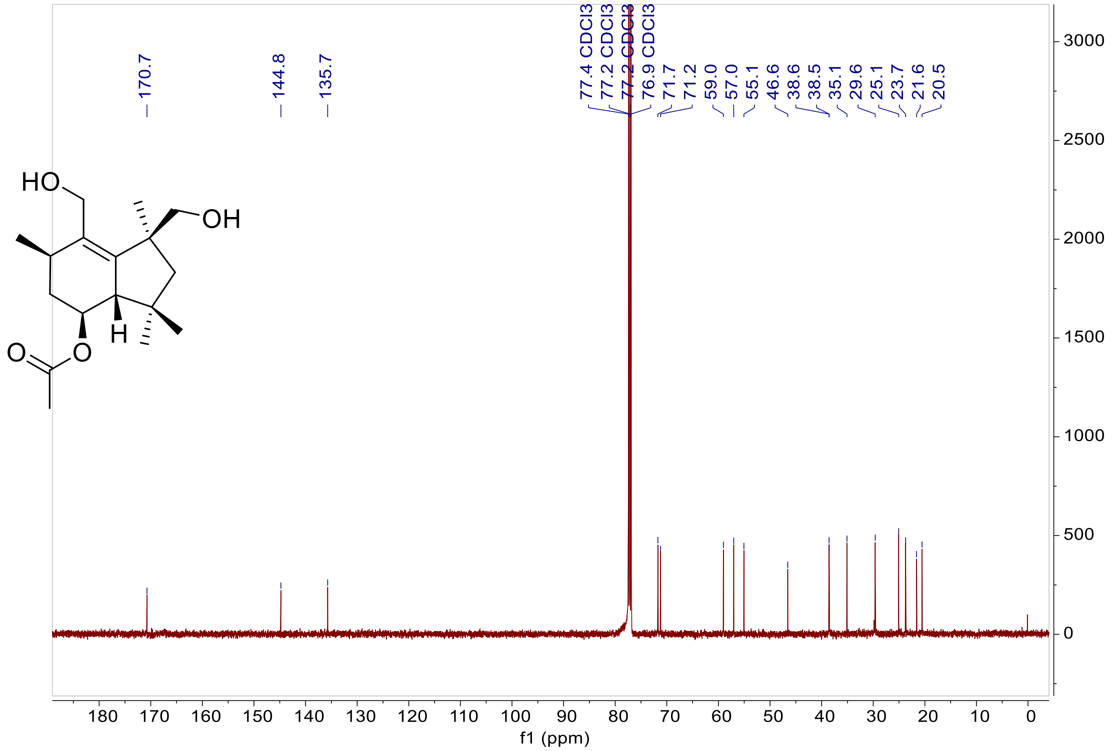

Figure S3. ^{13}C NMR spectrum (125 MHz, $CDCl_3$) of compound **1**.

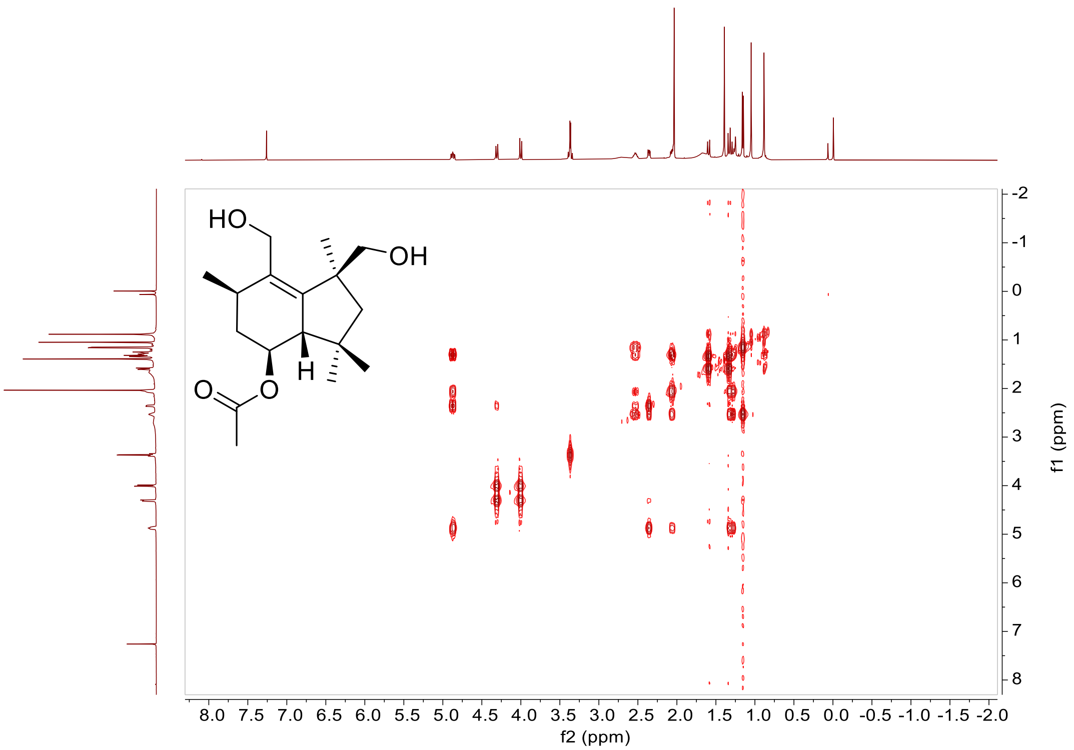

Figure S4. ^{1}H-^{1}H COSY spectrum of compound **1**.

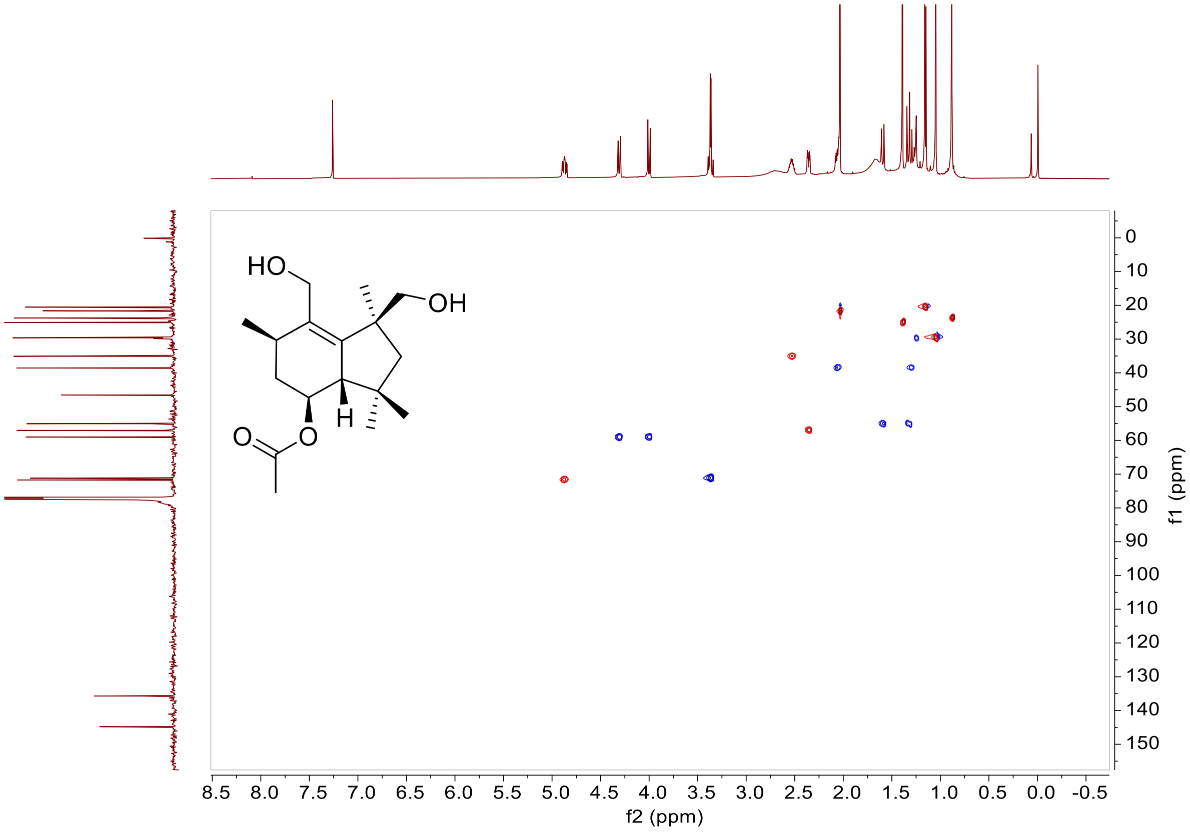

Figure S5. HSQC spectrum of compound **1**.

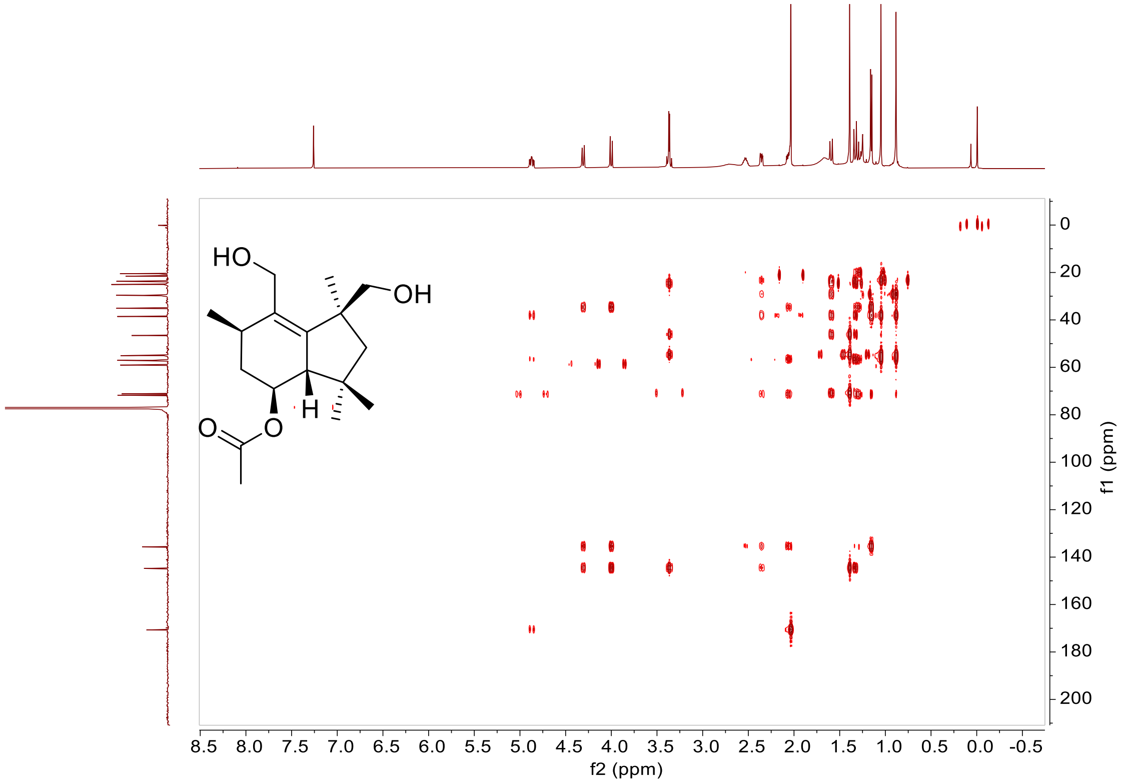

Figure S6. HMBC spectrum of compound **1**.

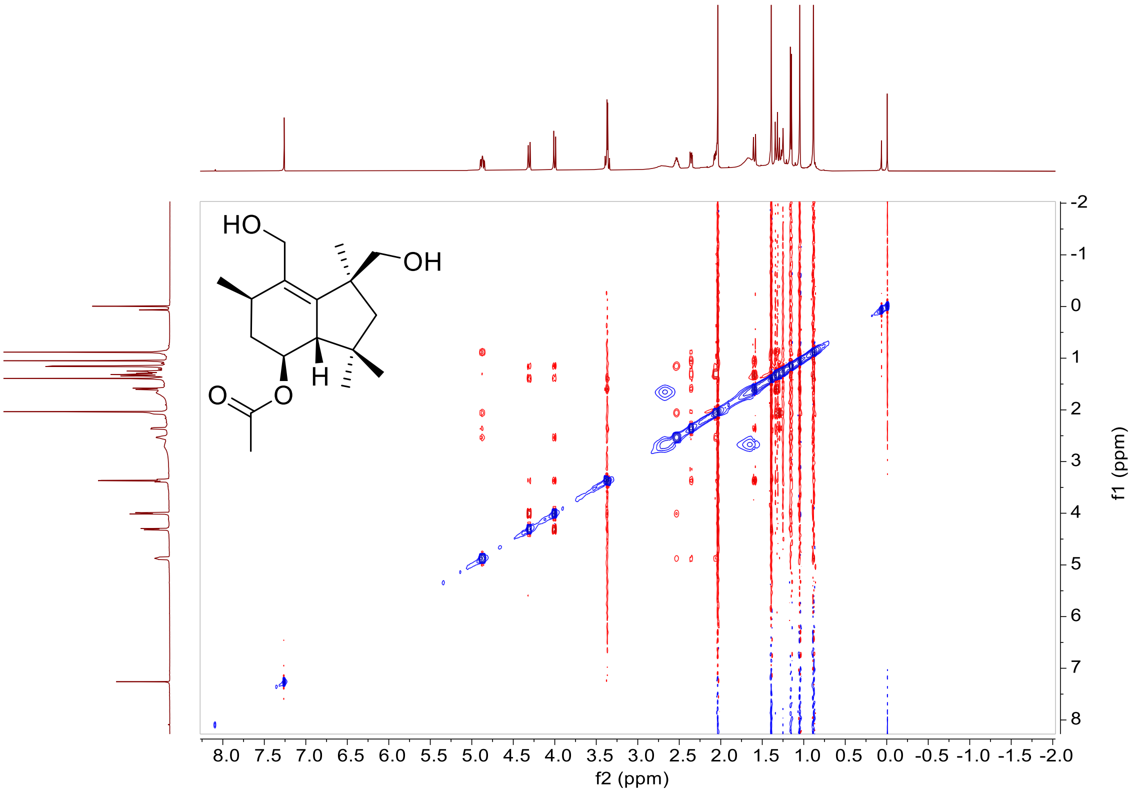

Figure S7. NOESY spectrum of compound **1**.

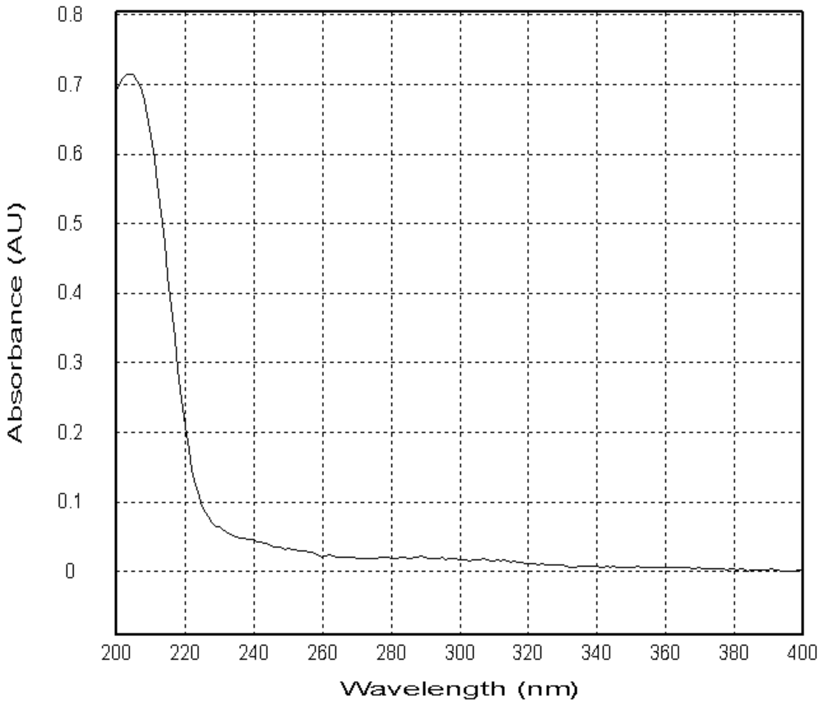

Figure S8. UV spectrum of compound **1**.

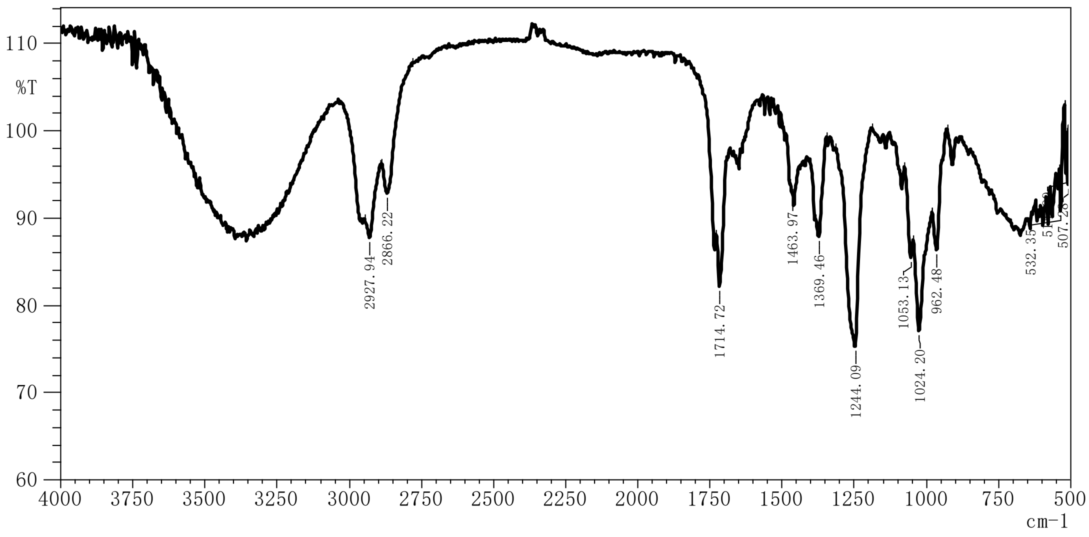

Figure S9. IR spectrum of compound **1**.
